# Case-Specific Focal Sensor Design for Cardiac Electrical Impedance Tomography

**DOI:** 10.3390/s22228698

**Published:** 2022-11-10

**Authors:** Chenke Zhang, Yu Wang, Shangjie Ren, Feng Dong

**Affiliations:** Tianjin Key Laboratory of Process Measurement and Control, School of Electrical and Information Engineering, Tianjin University, Tianjin 300072, China

**Keywords:** electrical impedance tomography, conformal transformation, sensor design, cardiac imaging

## Abstract

Electrical impedance tomography (EIT) is a non-invasive detection technology that uses the electrical response value at the boundary of an observation field to image the conductivity changes in an area. When EIT is applied to the thoracic cavity of the human body, the conductivity change caused by the heartbeat will be concentrated in a sub-region of the thoracic cavity, that is, the heart region. In order to improve the spatial resolution of the target region, two sensor optimization methods based on conformal mapping theory were proposed in this study. The effectiveness of the proposed method was verified by simulation and phantom experiment. The qualitative analysis and quantitative index evaluation of the reconstructed image showed that the optimized model could achieve higher imaging accuracy of the heart region compared with the standard sensor. The reconstruction results could effectively reflect the periodic diastolic and systolic movements of the heart and had a better ability to recognize the position of the heart in the thoracic cavity.

## 1. Introduction

Electrical impedance tomography is a non-invasive imaging method [1], which applies current or voltage excitation to electrodes at the boundary of an observation domain and uses the obtained electrical response signals to reconstruct the electrical conductivity distribution in the domain. Due to its low cost, portable equipment, high time resolution and lack of radiation, EIT has received extensive attention in biomedical imaging [2], and it has great potential application prospects and application value in the continuous monitoring of the functions of the human heart [3,4], lungs [5,6,7], brain [8,9], breast [10,11,12], abdomen [13,14] and other major organs.

As one of the most important organs of the human body, the contraction of the heart will cause changes in its volume, which in turn causes changes in its conductivity. Therefore, EIT has received extensive attention in the field of cardiac function monitoring [15]. Vonk-Noordegraaf et al. [16] calculated the cardiac stroke volume (SV) by monitoring the electrical conductivity changes in the heart region in the EIT image during the cardiac cycle. The experimental results proved that EIT is effective and repeatable for evaluating SV. In 2003, Fu Feng et al. [17] used EIT to image isolated animal hearts immersed in culture fluid and verified the feasibility of EIT to monitor cardiac function by simulating different filling states of the heart. It was pointed out that EIT images are sensitive to the electrical impedance changes in the cardiac chambers, which proves the potential application value of EIT in the imaging of the cardiovascular system. In 2015, Proenca et al. [18] pointed out that the electrical impedance changes in ventricles were mainly caused by the deformation of the left ventricle based on a single-sample dynamic simulation study. In 2019, Braun et al. [19] demonstrated that the SV changes in critically ill patients during fluid resuscitation can be monitored non-invasively by analyzing the synchronized impedance changes in the lung and heart. Therefore, EIT has been considered as one of the most promising cardiac-function-monitoring methods.

**Figure 1:**
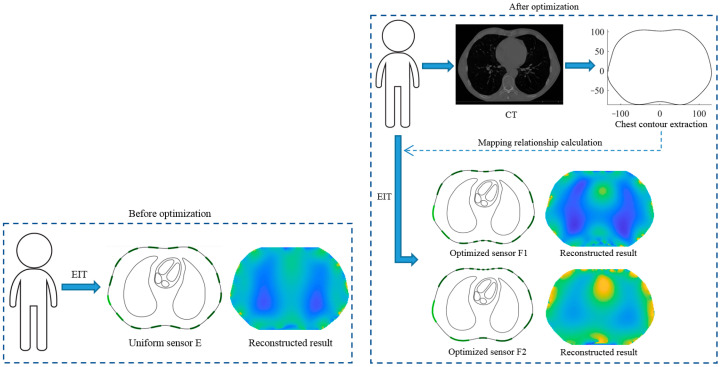
Thoracic EIT measurement process and simulation results before and after optimization.

Although there are some studies on cardiac EIT, due to its ill-posedness and non-linearity, the spatial resolution of cardiac EIT is relatively low. In most practical applications of EIT, the electrodes are always with the same size and placed evenly on the boundary of the observation domain such as the chest. However, scholars have proved that with this electrode arrangement, the reconstruction quality of the inclusions near and far from the electrode is relatively poor, but this problem can be effectively improved by optimizing the sensor array [20]. In this paper, a known chest boundary shape was considered, e.g., one extracted from a pre-collected lung CT image. Aiming at the long-term functional imaging of the human heart, when the heart is continuously detected, the conductivity changes are concentrated in a sub-region of the thoracic domain. Starting from this point, two optimized sensor designs were proposed based on the conformal transformation theory, which took the center of the chest cavity and the center of the heart as the mapping centers, respectively, to map the standard sensor defined on the unit circle corresponding to the chest cavity. Figure 1 shows the EIT measurement process and results of the human chest before and after sensor optimization. The thoracic contours of different cases were different, and the sensor optimization method proposed in this paper could calculate the specific mapping relation *f* for each case, and then obtain the case-specific optimized sensor array. By optimizing the sensor arrangement, the electric field distribution was changed, thereby improving the sensitivity and spatial resolution of cardiac EIT at the heart region. The calculation results of the two focused sensors F1 and F2 were compared with those of a uniform sensor E, and the reconstruction results were evaluated qualitatively and quantitatively.

## 2. Methodology

### 2.1. Principle of EIT

By arranging a certain number of electrodes on the boundary of the observation field, the electrical response data of the observation field was obtained according to a specific data collection mode, and then the reconstruction algorithm was used to calculate the electrical conductivity distribution in the field. This is the measurement principle of EIT. The Sheffield adjacent current stimulation and adjacent voltage measurement mode is one of the most famous data acquisition modes in EIT. Each time a pair of adjacent electrodes is excited, voltage measurements are made between other adjacent pairs of electrodes except for the driven electrodes. This process is repeated until all electrodes are excited.

The EIT method mainly focuses on the solution of forward and inverse problems. The forward problem is to calculate the boundary voltage according to the given conductivity distribution. It can be formulated as an elliptic partial differential equation with mixed boundary conditions and can be solved by the boundary element method (BEM) [21] or the finite element method (FEM) [22]. By considering the measurement noise, the voltage data of EIT can be defined as:

(1) η=V(σ)+ε

where *V* represents the calculated voltage, *σ* is the conductivity, *η* is the measured voltage and *ε* is the noise.

### 2.2. Inverse Problem

The inverse problem is to estimate the conductivity *σ* from the measured boundary voltage *η*. Following a different imaging framework [1], the voltage observation Equation (1) can be rewritten as:

(2) y=Jx+Δε

with *J* = *∂V/∂σ*, *x* = *σ* − *σ*_r_ and *y* = *η − η*_r_. The subscript *r* denotes the reference state. The sensitivity matrix *J* represents the Jacobian matrix, in which the boundary voltage changes with respect to the electric conductivity changes in the field. Different from (1), the new observation model (2) is linear, and the noise is suppressed by the subtraction operator.

Due to the ill-posedness of the EIT inverse problem, a small disturbance of the boundary measurement signal will cause a large change in the reconstruction parameters [23]. Regularization methods are often used to solve such ill-posed problems. Tikhonov regularization [24] is one of the most widely used regularization methods, which transforms Equation (2) into a least-squares problem with regularization constraints. Assuming the regularization factor is *λ*_1_, the minimization objective function is:

(3) $$∥Jx-y{∥}_{2}^{2}+\lambda_{1}∥x{∥}_{2}^{2}$$

For different regularization methods, by introducing different prior information, the constraints will have different forms. The Newton one-step error reconstructor (NOSER) algorithm [25] is solved by a one-step Newton method. It assumes that the initial conductivity distribution set by the Newton method is close enough to the true distribution, and then it only needs to iterate once to obtain an approximate solution. The solution of the NOESR algorithm is in the following form:

(4) $$x={\left(J^{T}J+\lambda_{2}G\right)}^{-1}Jy$$

where *G* is the diagonal matrix composed of the main diagonal elements of *J^T^J*. *TV* regularization [26] is a classic block-constrained EIT image reconstruction algorithm with good marginal preservation. Its minimization objective function is:

(5) $$∥Jx-y{∥}_{2}^{2}+\lambda_{3}\int_{\Omega }\sqrt{|\nabla x|+\theta }d\Omega$$

In order to ensure the differentiability of the function, *θ* is defined as a small constant. This study selected the values of the regularization factors *λ*_3_ and *θ* through empirical methods.

## 3. Modeling

### 3.1. Anatomical Model

**Figure 2:**
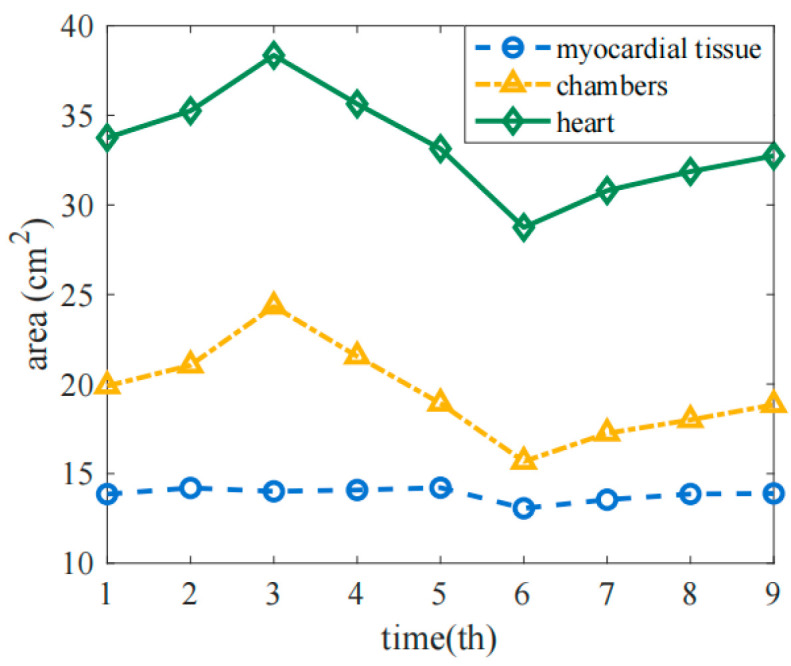
The change curve of the cross-sectional area of each heart chamber in a cardiac cycle.

Based on the dynamic image of the thoracic cavity cross-section obtained from the complete anatomy, nine images at nine moments in a cardiac cycle were intercepted and segmented. The obtained two-dimensional anatomical images of the thoracic region were binarized with the same resolution, the boundary was extracted, and the observation domain was divided to establish an anatomical model. Statistical analysis was performed on the cross-sectional area changes in the heart chambers obtained from the anatomical model. Figure 2 shows the cross-sectional area change curve of the heart chambers in a cardiac cycle, where the x-coordinate represents the order of nine moments selected in a cardiac cycle, and the y-coordinate represents the cross-sectional area of the heart. The results met the expected requirements. The area of myocardial tissue hardly changed during the cardiac cycle, while the diastolic-contraction movement of the entire heart caused significant changes in its area, and the trend was consistent with reality. It can be considered that this model could relatively accurately reflect the dynamic distribution of the heart in the thoracic region during a cardiac cycle. The total area of the thoracic region in the model was 407.5 cm^2^.

### 3.2. Boundary Element Simulation Model

The forward simulation model was established in EIDORS [27] and is as follows. Sixteen electrodes are placed on the unit circular boundary and the duty cycle is set to 50%. Two optimized thoracic fields and electrode arrays are obtained by mapping. Adjacent current excitation is adopted in the adjacent voltage measurement data acquisition mode. Each time an adjacent pair of electrodes is excited, the voltage between adjacent pairs of electrodes except for the driving electrode is measured, and this process is repeated until all electrodes are excited. In this study, the boundary conditions of the all-electrode model (CEM) were adopted, and a total of 208 voltage measurements were collected in one measurement period after all excitation sequences were completed. The numerical BEM [21] method was used to solve the forward problem. Referring to the chest simulation experiment conducted by Hamilton et al. [28] in 2017, the background conductivity was set to 0.3 S/m, the excitation frequency was set to 50 kHz and organs were added. The conductivity of the lungs, myocardial tissues and blood were determined in the IT’IS parameter database [29] and were set to 0.1 S/m, 0.2 S/m and 0.7 S/m, respectively. Available online: www.itis.ethz.ch/database (accessed on 1 November 2021).

In this research, the FEM method was used to calculate the inverse problem. The thoracic field was divided by square grids with the same area, and 2096 pixels were obtained. Assuming that the conductivity values in each grid were the same, the difference-imaging method was selected to reconstruct the image of the heart and lungs to reduce the measurement error.

### 3.3. Sensor Optimization

A standard EIT sensor uses a uniform electrode array to measure the observation field. However, in some applications, such as dynamic monitoring and imaging during the cardiac cycle, only the local area of the thoracic cavity is concerned. In order to improve the applicability of EIT and the image reconstruction quality of the heart region, two optimization models of the EIT sensor array based on conformal transformation were proposed.

**Figure 3:**
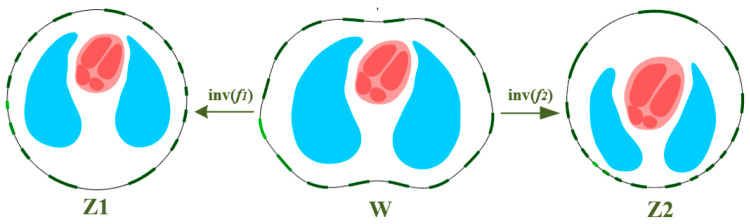
Chest anatomy image extraction and mapping.

The Schwarz-Christoffel (SC) transformation [30] can realize the conformal mapping from a thoracic domain W to a unit circle domain *Z* with the same physical characteristics. The SC transform calculates the mapping relationship of the boundary points between the two fields, so specific mapping results can be calculated for chest contours of different shapes, and this realizes one-to-one correspondence between the points (*x*, *y*) in the thoracic cavity and the points (*u*, *v*) in the circle area through the mapping relationship. The SC formula for the mapping f is:

(6) $$f\left(z\right)=f\left(z_{0}\right)+c\int_{z_{0}}^{z}\overset{n}{\underset{j=1}{\prod }}{\left(\delta -z_{j}\right)}^{\frac{\alpha_{j}}{\pi }-1}d\delta$$

where *z_j_* is the *j*-th boundary point of the original domain, *α_j_* is the internal angle of the *j*-th boundary point after mapping and *δ* is the discrete point on the boundary of domain *z*. In the formula, *z*_0_ and *c* are both complex constants, and for *j* = 1,2, …, *n*, the following formula always holds:

(7) $$w_{j}=f(z_{j})$$

where *w_j_* is the *j*-th boundary point of the mapped domain. This article used a chest CT image from the TCIA database, and the thoracic section between the fourth and fifth ribs was selected to establish a two-dimensional thoracic model. Since the heart is static in the CT image, the thoracic cavity boundary was extracted from the CT image, and the lungs and dynamic hearts segmented from the complete anatomy were placed into the thoracic cavity to obtain a complete two-dimensional thoracic model W with uniform sensors, as shown in Figure 3. The circular regions Z1 and Z2 are the results of W through two inverse mappings inv(f1) and inv(f2). It can be seen that the electrodes and the inclusions were mapped accordingly.

In the two mapping results shown in Figure 3, the electrodes were densely distributed near the lungs, while our detection object was the heart area; it did not match our goal. In order to improve the spatial resolution of the heart area, two sensor optimization schemes were proposed.

**Figure 4:**
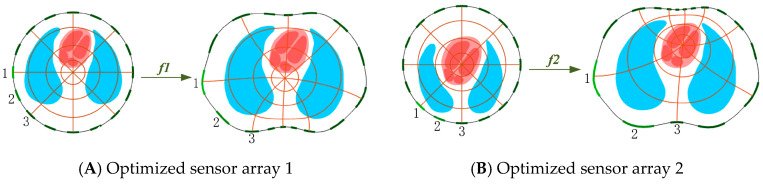
Two mapping processes, taking the center of the thoracic region as the mapping origin (**A**) and taking the center of the heart region as the mapping origin (**B**).

The point (*x*, *y*) of the thoracic region could be mapped to the unit circle region (*u*, *v*), and the point of the unit circle could also be mapped to the thoracic region. By mapping the evenly distributed sensors in the unit circle to the thoracic region, two optimized sensor models were obtained. Figure 4 shows the mapping process, including three circles centered at the mapping origin and eight evenly spaced radii, where all intersections are orthogonal. Model A took the center of the thoracic region as the mapping origin. The optimized sensor array was densely distributed on the longitudinal line of the heart position, and the electrode spacing on both sides gradually increased. Model B used the center of the heart area as the mapping origin to optimize the sensor array, ensuring that each electrode contributed the same to the heart, and after optimization, the electrodes near the heart area were denser than in model A. Both mapping methods could improve the sensitivity near the heart region and theoretically improve the reconstruction accuracy of the heart.

## 4. Result Analysis

### 4.1. Sensitivity Calculation

**Figure 5:**
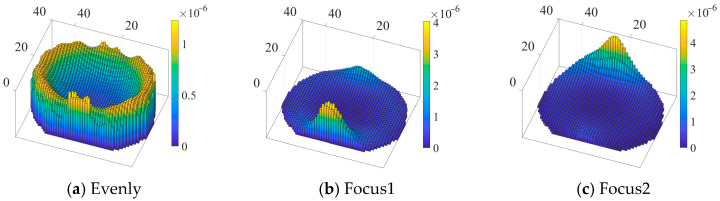
Sensitivity distribution of (**a**) uniform electrode array, (**b**) optimized array1 and (**c**) optimized array2.

Figure 5 shows the sensitivity distribution of the thoracic region E under the uniform electrode array and the sensitivity distributions of the fields F1 and F2 after optimizing the electrode arrays. The sensitivity value represents the change in the boundary voltage measurement value caused by the change in the conductivity of each pixel [31]. This study used the perturbation method to obtain the sensitivity matrix, and the formula is:

(8) $$J_{ij}=\frac{v_{a_{ij}}-v_{b_{i}}}{\beta },i=1,2,\ldots ,208;j=1,2,\ldots ,2096$$

where $v_{b_{i}}$ is the measured value of the *i*-th boundary potential of the empty field, $v_{a_{ij}}$ represents the measured value of the *i*-th boundary potential when disturbance is applied to the *j*-th grid point and *β* is a small disturbance, which was set as 0.1. It can be seen that the sensitivity distribution before optimization was relatively uniform, but the sensitivity value was low, which was not conducive to high-precision imaging. Table 1 compares the mean and standard deviation of the sensitivity distribution of the heart region under the three models. The average sensitivity of the optimized electrode array increased, which meant that the optimized sensor could more easily detect the conductivity changes near the heart region, and the spatial resolution of the heart region was improved.

### 4.2. Boundary Potential

**Figure 6:**
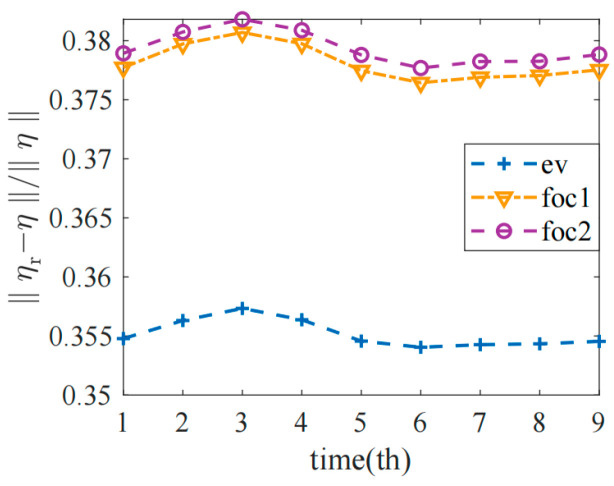
Boundary potential changes in the standard model and the optimized models during a cardiac cycle.

Figure 6 shows the comparison of the boundary potential between the uniform model and the optimized models, where *η_r_* represents the measured boundary voltage of the target field and *η* represents the measured value of the empty field. In order to reduce the effect of model error, the relative changes in the two norms of *η_r_* and *η* were calculated at nine moments in a cardiac cycle, and the results of the third models were compared.

As shown in the figure, the optimized models F1 and F2 had higher boundary potentials, indicating that they were more sensitive to the conductivity changes in the observation field, which was consistent with the sensitivity results in Section 4.1, indicating that the imaging accuracy of the observation field could be effectively improved after sensor optimization. However, the line trend of the two optimized models was the same as that of the uniform sensor model E, which proved that the proposed optimization method was reasonable and feasible.

### 4.3. Analysis of Reconstruction Results

The first column of Table 2 shows the two-dimensional model for nine moments in a cardiac cycle. After adding a 40 dB signal-to-noise ratio Gaussian white noise to the boundary measurement data of the empty field and the object field, the differential reconstruction results of the three electrode arrays and different imaging methods were obtained. The noise signal calculation equation is in the following form:

(9) $$sig_{n}=noi\times \frac{v_{i}}{∥noi∥\times SNR}$$

where *v_i_* is the calculated value of the boundary voltage and *noi* is the generated random array of standard normal distribution with the same dimension as *v_i_*.

In Table 2, columns 2–4 used the TV imaging algorithm, where the model order is E, F1 and F2. Columns 5–7 represent the Tikhnonov regularization reconstruction results and the last three columns are under the NOSER algorithm. For the three reconstruction algorithms used, the TV regularization algorithm had good margin preservation and had a good reconstruction effect for the size and position of the heart. As a non-iterative algorithm, Tikhnonov has a faster imaging speed, and the boundary resolution of the region was lower due to the smooth prior information introduced. In the results obtained by the NOSER algorithm, the pixel contrast was larger, and the size of the imaging target was smaller.

It is known that in the established 2D heart model, t3 is the end diastole of the heart, the cross-sectional area of the heart is the largest, t6 is the end systole of the heart, and the cross-sectional area of the heart is the smallest. Comparing the imaging results of the uniform sensor model E and the two optimized models F1 and F2, it can be seen that the uniform electrode model E reflected the beating process of the heart more in its position change, and due to the imaging artifacts near the electrode, the end-systolic heart reconstruction result was larger, contrary to reality. In comparison, the optimized results could more intuitively reflect the cardiac contraction process through the change in pixel value. The simulation models after optimizing the electrode array were more accurate for the position of the heart and more sensitive to the periodic changes in the heart. Regarding a periodic change in the heart, the blood conductivity was constant, but the periodic beating of the heart caused its cross-sectional area to change. Among all the results shown in Table 2, the second optimization method had the best results under the TV algorithm, which could clearly reflect the cyclical changes in the heart.

The correlation coefficient (*CC*), relative image error (*RE*), position error (*PE*) and shape deformation (*SD*) between the true distribution and the reconstructed image of the heart region were used as evaluation indicators. These evaluation indicators are defined as follows:

(10) $$CC=\frac{\sum_{i=1}^{n}\left(\alpha_{i}-\bar{\alpha }\right)\left(\alpha_{ki}-{\bar{\alpha }}_{k}\right)}{\sqrt{\sum_{i=1}^{n}{\left(\alpha_{i}-\bar{\alpha }\right)}^{2}\sum_{i=1}^{n}{\left(\alpha_{ki}-{\bar{\alpha }}_{k}\right)}^{2}}}$$

(11) $$RE=\frac{\alpha_{k}-\alpha }{\sqrt{l_{\alpha }}}$$

(12) $$PE=\sqrt{{\left(\alpha_{kx}-\alpha_{x}\right)}^{2}+{\left(\alpha_{ky}-\alpha_{y}\right)}^{2}}$$

(13) $$SD=\frac{\left\|\alpha_{k}-\alpha \right\|}{\left\|\alpha \right\|}$$

where *α_k_* is the binary vector of the conductivity distribution after reconstruction, where 70% of the maximum element value in the vector was set as the threshold [32], *α* is the binary vector of the original conductivity distribution and *α_i_* is the *i*-th element in *α*, and *α_ki_* is the *i*-th element in *α_k_*. *l_α_* represents the length of the vector *α*, *α_kx_* represents the x-coordinate of the centroid of the reconstructed image and *α_ky_* represents the y-coordinate of the centroid of the reconstructed image. Similarly, *α_x_* is the x-coordinate of the centroid of the original image and *α_y_* is its y-coordinate.

**Figure 7:**
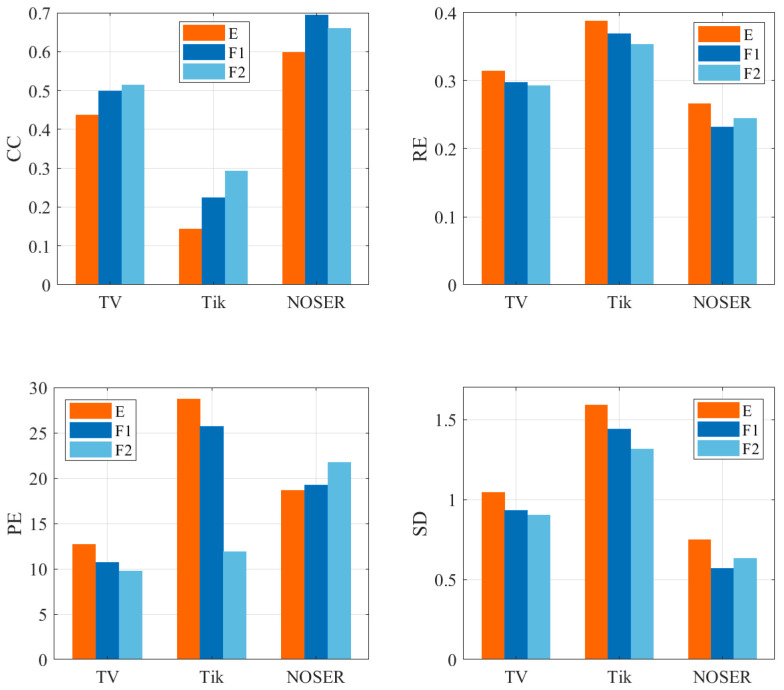
Quantitative analysis of the reconstruction results.

Figure 7 calculates the quantitative index of the reconstructed image of the three models under different algorithms at a certain cardiac cycle time. The low value of RE, PE, SD and the high value of CC signify that the result was good. Figure 7 shows that the reconstruction result of the heart area after the electrode array was optimized had a higher correlation and lower image error compared with its original image. Consistent with the imaging results, the quantitative indicators of F2 under the TV algorithm were the best, and F1 also obtained good quantitative results compared to E, which proves that optimizing the sensor array was beneficial in achieving a high-precision reconstruction of the heart region.

### 4.4. Average Cardiac Impedance

Considering that in the reconstructed image the systolic and diastolic movements of the heart in a heartbeat cycle were reflected by the changes in the reconstructed pixel values, in order to more intuitively describe the dynamic process of a heartbeat cycle, an EIT-based heart volume evaluation index ACI (average cardiac impulse) was proposed, which represented the average conductivity value in the heart region in the reconstructed image. The reconstructed pixels in the heart area were extracted, and the average value was calculated hoping to match the periodic change trend in the blood stored in the heart, which was represented by the change in the cross-sectional area of the heart. With the addition of noise with a signal-to-noise ratio of 40 db, the reconstruction distribution of the heart area at nine times under the three electrode arrangements was measured 10 times, and 3 × 9 × 10 data points were obtained. Since only the change trend was concerned here, the calculated mean discrete data and the true cross-sectional area change in one cardiac cycle were normalized. The ACI was normalized by the following formula:

(14) $${\tilde{A}}_{t}=\frac{A_{t}-\min\left(A_{t}\right)}{\max\left(A_{t}\right)-\min\left(A_{t}\right)}$$

**Figure 8:**
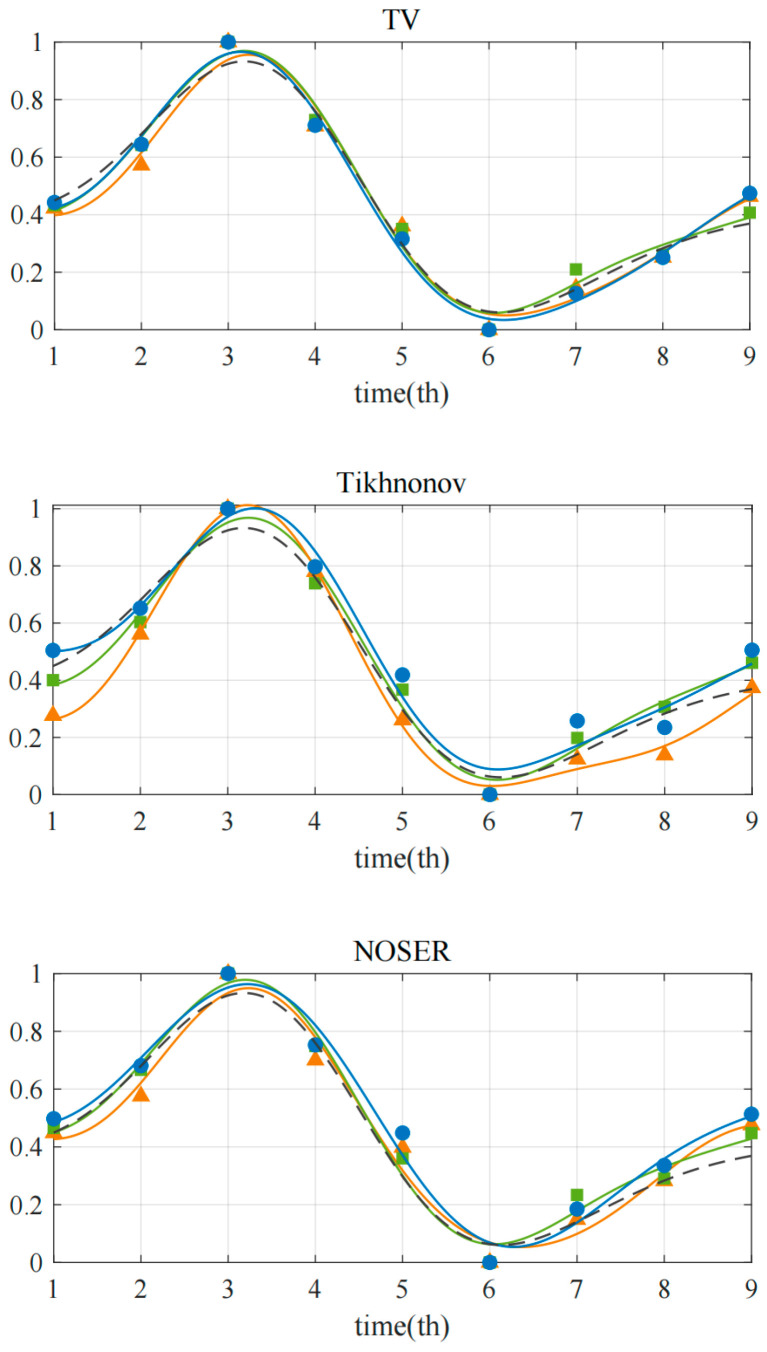

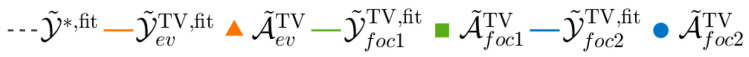
Comparison of the normalized reconstruction pixel values of the heart region under three models and the real area trend.

Curve fitting was performed on the processed data points to simulate a complete cycle. A smoothing spline was used to fit the data of nine moments in a cardiac cycle to obtain the final curve. The results are shown in Figure 8, where ${\tilde{A}}_{t}^{*}$ represents the real heart area in the original field at time t after normalization, which is smoothly fitted to ${\tilde{A}}^{*,fit}$ as shown by the dotted line in the figure.$\;A_{ev}^{TV}$ represents the mean value of the reconstructed conductivity in the heart region when model E adopted the TV algorithm, and ${\tilde{A}}_{ev}^{TV}$ was obtained after normalization. ${\tilde{A}}_{ev}^{TV,fit}$ represents the dynamic change in the ACI in a heartbeat cycle after the smooth fitting of ${\tilde{A}}_{ev}^{TV}$. In Figure 8, the dotted line represents the change in the original cross-sectional area of the heart, the triangle represents the numerical point obtained by normalizing the ACI value of the uniform sensor model, the square represents the optimized model F1, and the circle represents the optimized model F2. The results showed that the optimized models reflected the systolic changes in the heart more accurately, which was most obvious under the Tikhnonov algorithm, in which the model E had two error trends at time 1 and time 8. Comparing the two proposed optimization methods, it can be seen that the curve trend of the model F1 was more in line with the change in the original cross-sectional area, which could better reflect the periodic beating of the heart. In addition, considering that the first optimization method used the center of the thoracic cavity as the mapping center and did not require the specific location of the heart, the calculation was simpler and more convenient, and the applicability was thus wider.

## 5. Experimental Result

**Figure 9:**
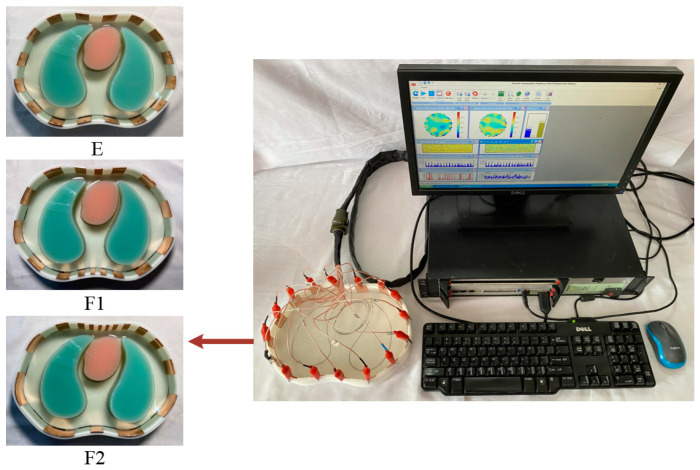
Experiment equipment consisting of the designed sensor and data acquisition system.

The two proposed electrode optimization methods were experimentally validated using the EIT system designed by Tianjin University [33]. Figure 9 shows the experimental system, including the designed sensor and a 16-channel high-speed parallel data acquisition system. The chest model was made of resin material by 3D printing. For the two proposed electrode array optimization strategies, the sensor part used an inner ring made of resin material and an electrode ring composed of copper electrodes. Three electrode arrays before and after optimization corresponded to three electrode rings, respectively, the inner diameter of the electrode ring was the same as the simulation and the outer diameter was the same as the inner diameter of the thoracic cavity model, which could be embedded in the thoracic cavity model. In the experiment, different electrode arrays could be selected by simply replacing the different electrode inner rings.

The lung and heart phantom models at different times were made of different concentrations of NaCl and agar powder. The conductivity settings of each phantom were consistent with the simulation. The background conductivity was set to 0.3 S/m, and the lung phantom was 0.1 S/m. Due to the complicated changes in the volume of each chamber of the heart during the cardiac cycle (shown as cross-sectional area changes in two dimensions), the myocardial tissue and the atria and ventricles were considered as the whole heart in the experiment, and the conductivity of the cardiac phantom was set to a blood conductivity of 0.7 S/m.

Experiments were carried out on the thoracic model under three electrode arrays before and after optimization, in which nine heart moments were measured under each electrode distribution, and a total of 3 × 9 groups of object field boundary voltage values and three groups of empty field voltage values were obtained.

### 5.1. Measurement Voltage Analysis

**Figure 10:**
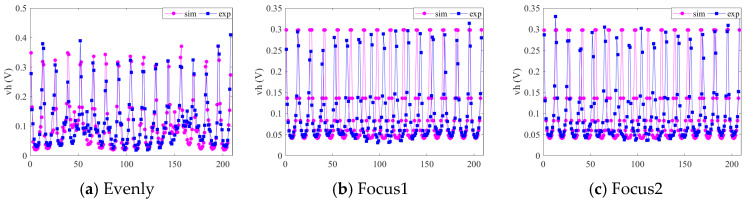
Comparison of experimental and simulated voltage waveforms.

The experimental voltage measurement data of the empty field under the three electrode arrays were scaled appropriately, and the waveform was compared with the simulation voltage waveform. The results are shown in Figure 10. Since the experiment used a parallel system, the difference in the different acquisition channels led to a difference in the voltage measurement peak under different excitations, so there were reasonable differences between the simulation results and the measurement results. However, it can be seen that the trends of the experimental and simulation data results were consistent, which verified the reliability of the experimental results. Since the electrode arrays of models F1 and F2 were mapped from a uniform array of circular fields, the simulated voltage waveforms were standard U-shaped.

### 5.2. Analysis of Experimental Results

For the nine moments in a cardiac cycle, different imaging algorithms were used to calculate the inverse problem, and the results are shown in Table 3. It is known that t3 is the end-diastole period and t6 is the end-systole period. From the imaging results, the uniform sensor model E could not directly reflect the periodic beating process of the heart. It was more reflected in the position change, but the heart position also had a large deviation from the original position. The optimized F1 and F2 models reconstructed the heart position more accurately and had more obvious periodic changes. Compared with NOSER, the TV and Tikhnonov algorithms obtained better results. The systolic and diastolic process of the heart was directly reflected in the change in the pixel values. The pixel value of the heart part was significantly higher at t3, while t3–t6 is the contraction process of the heart, which showed a gradual decrease in the pixel value in the imaging results. The results obtained by the F2 optimization model using the TV algorithm were optimal, the description of the position and size of the heart was very accurate and the periodic beating process of the heart could be clearly reconstructed.

**Figure 11:**
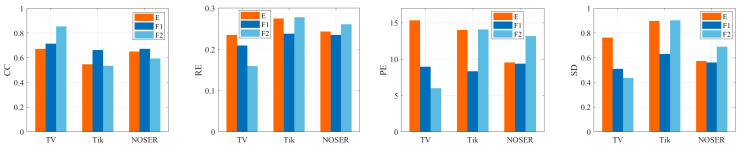
Quantitative analysis of the experimental results.

The quantitative indicators RE, CC, PE and SD were still used to evaluate the reconstruction results obtained in the experiment. The original heart boundary was used as the binarization standard to calculate the pixel value vector of the original distribution, and the reconstructed binarized vector was calculated with a pixel threshold of 70% as the standard. Taking the average value of RE and CC at nine times under each array and each algorithm, from the results in Figure 11, the proposed model F1 was better than the uniform electrode array under the three algorithms, with higher CC values and lower RE, PE and SD values. F2 performed best under the TV algorithm, while in Tikhnonov and NOSER, the quantitative index results were mediocre due to the inaccurate reconstruction of the cardiac position. Considering the slow imaging speed of TV as an iterative algorithm, the first optimization model F1 was more preferable.

**Figure 12:**
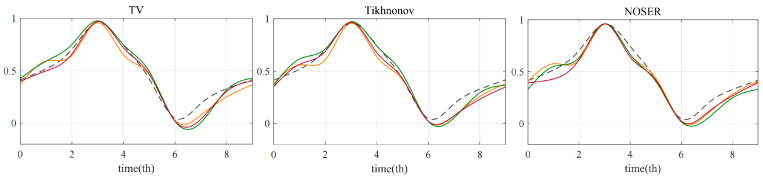
Reconstruction curve of pixel values in cardiac region under each algorithm.

The reconstructed ACI value was calculated to reflect the periodic beating process of the heart more intuitively. First, the reconstructed pixel matrix at each heart time was normalized. Considering that the heart boundary is not easy to obtain in a real situation, the ACI of the experimental results was calculated by the threshold method, and pixel values greater than 0.8 were extracted and the average value was taken. In order to simulate a complete heartbeat cycle, the value at t9 was also regarded as t0, and the data points at the ten times were fitted as a curve, which was compared with the change curve of the heart cross-sectional area of the original model. The results are shown in Figure 12, where the dotted line represents the original change. The three graphs show that the heart region reconstruction curves of the three electrode arrays under the three algorithms could determine the end-diastolic period t3 and the end-systolic period t6. However, for the whole heart-beating process, if the trend of each time point is considered, the proposed optimized models had more accurate results, while the model E was relatively poor, indicating that the optimized sensor array could effectively improve the reconstruction accuracy of the heart region.

## 6. Conclusions

This research was based on the human body’s two-dimensional thoracic cavity model. In order to improve the accuracy of the image reconstruction of the heart region and visualize the heart-beating process, two sensor optimization designs were proposed. By selecting different mapping centers, the uniformly distributed electrodes in the unit circle field were mapped based on the conformal transformation to obtain two optimized sensor models. The proposed optimization method was verified by simulation and experiments. After testing the reconstruction results of the different reconstruction algorithms, the TV regularization algorithm performed the best, and quantitative indicators proved that the optimized models had a higher sensitivity distribution, higher image correlation and lower image error in the heart region. Taking all factors into consideration, model F1 was preferable because it had better indicators in the fast-imaging algorithm, and the mapping center could be directly determined by the thoracic boundary, which could make the optimization process simpler. The simulation and experimental results showed that the proposed method of optimizing the sensor achieved the purpose of improving the spatial resolution of the heart region.

## Figures and Tables

**Table 1:** Comparison of mean and standard deviation of sensitivity distributions of the heart region under the three models shown in Figure 4.

	Avg (×10^−7^)	Std (×10^−7^)
Evenly	4.18	2.05
Focus1	4.76	2.63
Focus2	5.10	3.42

**Table 2:** Image reconstruction results from proposed optimized sensors and uniform sensors under different algorithms.

	Phantoms	ev	foc1	foc2	ev	foc1	foc2	ev	foc1	foc2
t1	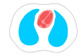	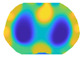	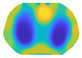	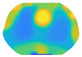	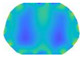	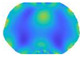	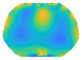	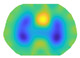	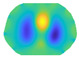	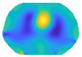
t2	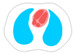	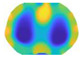	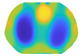	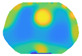	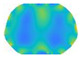	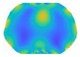	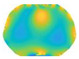	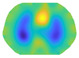	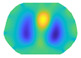	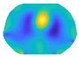
t3	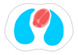	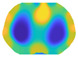	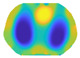	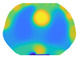	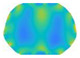	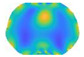	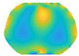	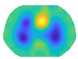	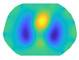	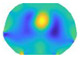
t4	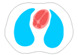	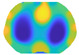	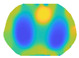	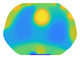	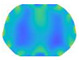	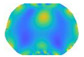	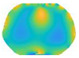	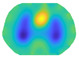	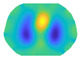	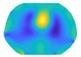
t5	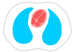	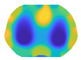	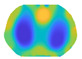	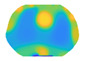	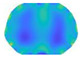	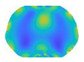	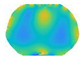	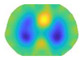	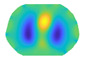	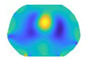
t6	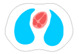	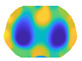	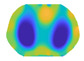	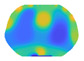	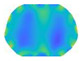	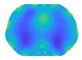	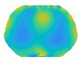	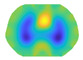	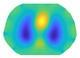	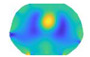
t7	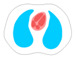	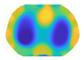	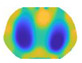	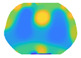	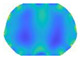	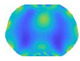	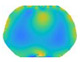	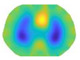	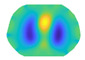	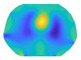
t8	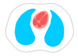	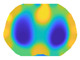	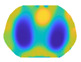	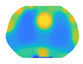	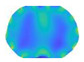	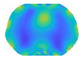	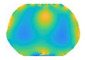	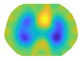	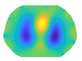	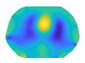
t9	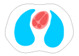	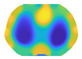	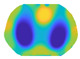	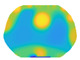	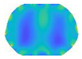	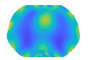	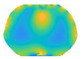	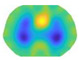	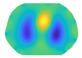	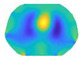

**Table 3:** Image reconstruction results from proposed optimized sensor and uniform sensor under different algorithms.

	Phantoms	ev	foc1	foc2	ev	foc1	foc2	ev	foc1	foc2
t1	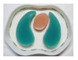	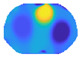	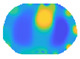	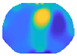	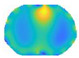	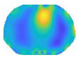	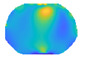	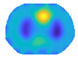	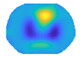	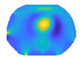
t2	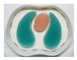	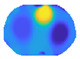	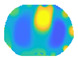	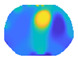	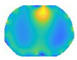	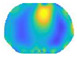	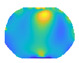	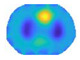	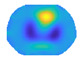	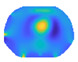
t3	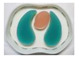	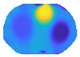	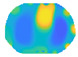	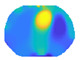	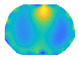	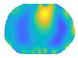	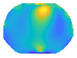	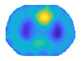	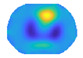	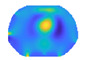
t4	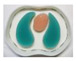	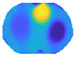	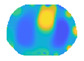	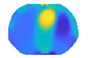	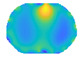	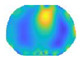	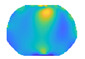	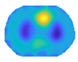	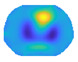	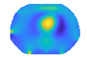
t5	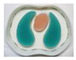	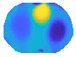	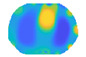	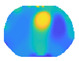	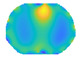	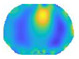	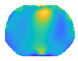	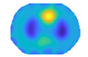	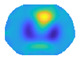	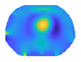
t6	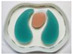	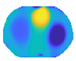	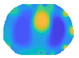	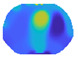	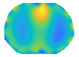	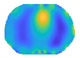	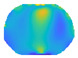	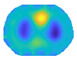	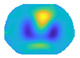	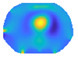
t7	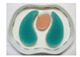	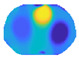	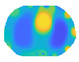	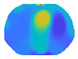	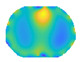	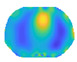	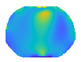	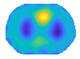	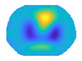	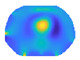
t8	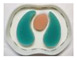	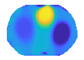	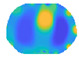	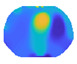	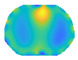	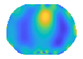	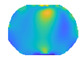	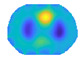	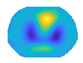	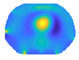
t9	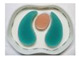	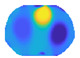	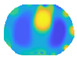	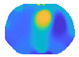	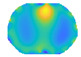	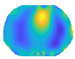	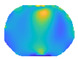	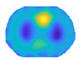	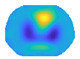	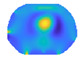
